# The long non-coding RNA MALAT1 regulates intestine host-microbe interactions and polyposis

**DOI:** 10.3389/fcell.2023.1168693

**Published:** 2023-05-30

**Authors:** Tianyun Long, Juan E. Hernandez, Shengyun Ma, Scarlet Steele, Claire Luo, Yuxin Li, Qinghong Xie, Francesca Telese, Bing Zhou, Wendy Jia Men Huang

**Affiliations:** ^1^ Department of Cellular and Molecular Medicine, University of California San Diego, La Jolla, CA, United States; ^2^ Department of Psychiatry, University of California San Diego, La Jolla, CA, United States; ^3^ State Key Laboratory of Stem Cell and Reproductive Biology, Institute of Zoology Chinese Academy of Sciences, Beijing, China; ^4^ Beijing Institute for Stem Cell and Regenerative Medicine, Beijing, China

**Keywords:** long non-coding RNA, MALAT1, intestinal cancer, intestine microbes, intestine epithelial cells

## Abstract

The long non-coding RNA (lncRNA) Metastasis-associated lung adenocarcinoma transcript 1 (MALAT1) maintains the integrity of the intestinal epithelial barrier and regulates local inflammation. However, its influences on intestinal microbial communities and tissue susceptibility to cancer development remain unexplored. Here, we report that MALAT1 regulates host anti-microbial response gene expression and the composition of mucosal-associated microbial communities in a region-specific manner. In the APC mutant mouse model of intestine tumorigenesis, knocking out MALAT1 results in higher polyp counts in the small intestine and colon. Interestingly, intestine polyps that developed in the absence of MALAT1 were smaller in size. These findings highlight the unexpected bivalent role of MALAT1 in restricting and promoting cancer progression at different disease stages. Among the 30 MALAT1-targets shared by both the small intestine and colon, ZNF638 and SENP8 levels are predictive of colon adenoma patient overall survival and disease-free survival. Genomic assays further revealed that MALAT1 modulates intestinal target expression and splicing through both direct and indirect mechanisms. This study expands the role of lncRNAs in regulating intestine homeostasis, microbial communities, and cancer pathogenesis.

## Introduction

Intestinal epithelial cells (IECs) provide a physical barrier against microbes and facilitate local immune responses (Ali et al., 2020). Genetic mutations and microbial challenges that impair IEC functions contribute to gastrointestinal illnesses like inflammatory bowel disease and colorectal cancer (Zheng et al., 2014; Li et al., 2016; Li et al., 2017a; Zhu and Xie, 2020). However, our understanding of the molecular regulators of IECs remains incomplete. The evolutionarily conserved Metastasis-associated lung adenocarcinoma transcript 1 (MALAT1), also known as nuclear enrichment autosomal transcript 2 (NEAT2), is one of the most abundantly expressed long non-coding RNAs (lncRNAs) in IECs. Recent studies suggest that human MALAT1 has an important role in maintaining the integrity of the intestinal epithelial barrier and contributes to local inflammation. By sequestering target microRNAs, MALAT1 maintains the expression of apical junction complex proteins NUMB and CLDN11 (Li et al., 2021). In Crohn’s disease patients and the dextran sulfate sodium-induced mouse model of colitis, MALAT1 is downregulated (Li et al., 2021). In Ulcerative colitis patients, however, MALAT1 is upregulated (Zhu and Xie, 2020). However, the physiologic and pathologic functions of MALAT1 in the intestine epithelium under homeostasis and disease settings remain unclear.

Mechanistic studies on MALAT1 have been performed in the context of lung, breast, cervix, and esophageal cancers, where MALAT1 expression is often dysregulated (Chou et al., 2016; Gong et al., 2016; Li et al., 2017a; Westphalen et al., 2017; Wang et al., 2018). Cell culture studies suggest that MALAT1 is enriched in the nucleus and regulates gene expression at multiple levels (Guo et al., 2010; Wang et al., 2015; Yang et al., 2015; Wang et al., 2016; Xia et al., 2016; Stamato et al., 2017; Wu et al., 2017). For example, it can interact with transcription factors and chromatin remodelers such as PRC2 to regulate transcription, pre-mRNA splicing, and act as a sponge to sequestrate microRNAs (Ji et al., 2003; Tripathi et al., 2010; Salmena et al., 2011; Yang et al., 2011; Miyagawa et al., 2012; Engreitz et al., 2014; Gong et al., 2016; Luan et al., 2016; Chang and Hu, 2018). Yet, the extent to which these mechanisms contribute to MALAT1 function *in vivo* under homeostasis and disease settings remains unclear.

In this study, we report that murine MALAT1 regulates the transcription and alternative splicing of a subset of IEC genes involved in microbial responses through both direct chromatin recruitment and indirect mechanisms. Knocking out MALAT1 results in altered intestine microbial communities and increases susceptibility to developing polyps in the small intestine and colon. These findings highlight the unexpected tumor suppressor function of MALAT1 in intestine tumorigenesis and provide insights into the contributions of lncRNAs in regulating IEC functions and the discovery of new therapeutic targets for intestinal cancers.

## Results

### MALAT1 regulates the abundance and splicing of IEC genes involved in anti-microbial responses

**FIGURE 1:**
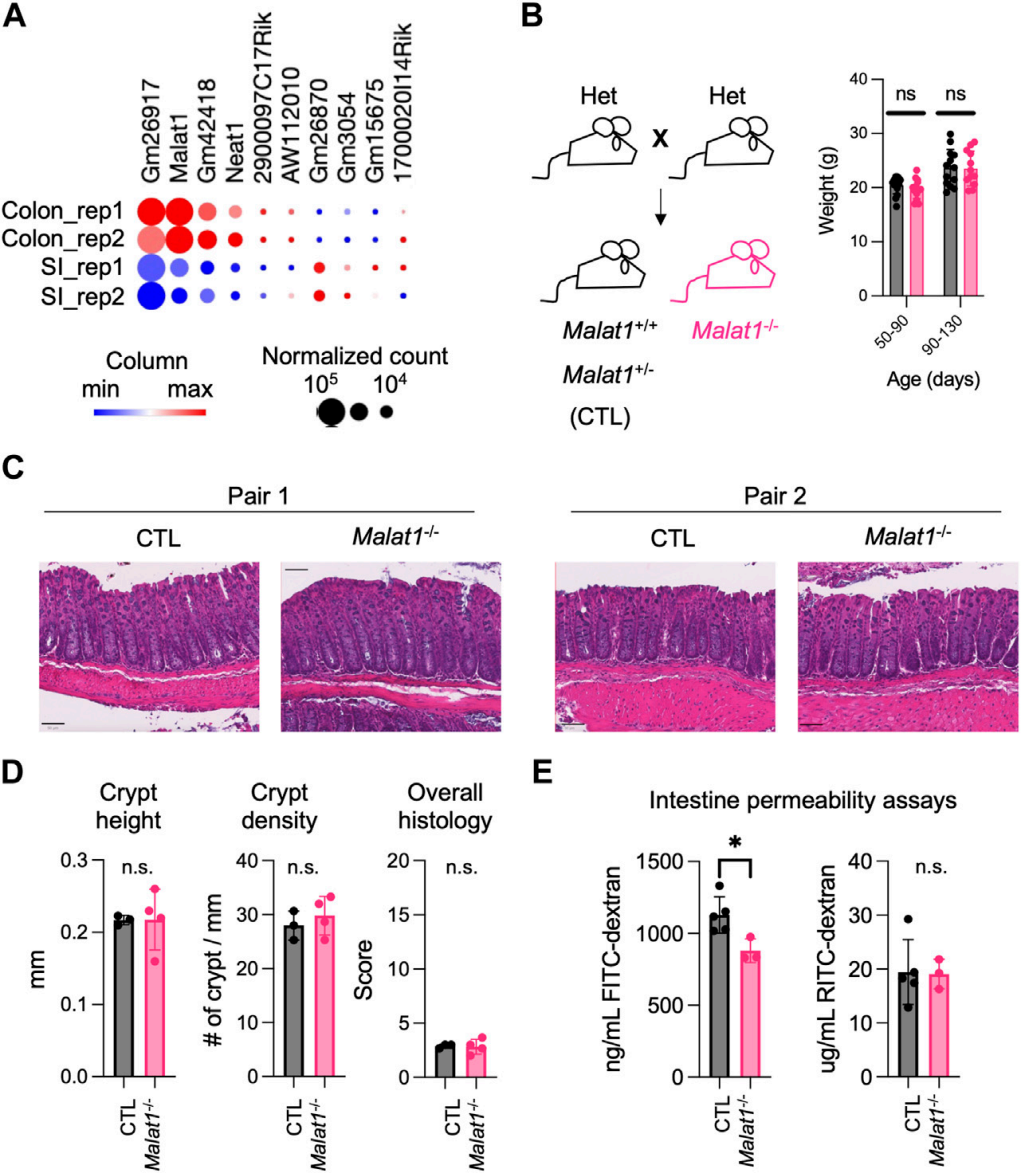
MALAT1 promotes protein-size macromolecule infiltration in the mouse intestine. **(A)**. Heatmap of top ten most abundantly expressed lncRNAs in the small intestine and colon epithelium isolated from two 8 weeks old wild-type male mice (GSE123881). Dot size indicates normalized read counts and color indicates relative expression levels of each gene among the four samples. Rep, biological replicate. **(B)**. Left: Breeding scheme for the generation of CTL (*Malat1* ^+/+^ or *Malat1* ^+/−^) and *Malat1* ^−/−^ cohoused littermates employed for this study. Right: Weight of CTL (*n* = 30) and *Malat1* ^−/−^ (*n* = 28) mice assessed at the indicated ages. Each dot represents the result from one mouse. ns: not significant (*t*-test). **(C)**. Representative H&E staining of colonic sections from two pairs of 12-week-old CTL and *Malat1* ^−/−^ female mice. **(D)**. Colon crypt height, density, and overall histology scores from CTL (*n* = 3) and *Malat1* ^−/−^ (*n* = 4) mice. n.s. not significant (*t*-test). **(E)**. Quantification of FITC-dextran and RITC-dextran in serum from CTL (*n* = 5) and *Malat1* ^−/−^ (*n* = 3) mice. Each dot represents the result from one mouse. * *p*-value<0.05, n.s. not significant (*t*-test).

MALAT1 is one of the most highly expressed long non-coding RNAs in both the small intestine and colon epithelium (Figure 1A). Previous studies suggest that MALAT1 can regulate gene expression at the transcriptional and post-transcriptional levels (Tripathi et al., 2010; Miyagawa et al., 2012). Based on these reports, we hypothesized that MALAT1 may contribute to intestine functions by regulating gene expressions in intestine epithelial cells. To test this possibility, we crossed the *Malat1* ^+/−^ mice to generate gender-matched and cohoused control (CTL, *Malat1* ^+/+^ and *Malat1* ^+/−^) and *Malat1* knockout (*Malat1* ^−/−^) littermates for our study. CTL and *Malat1* ^−/−^ littermates were born in Mendelian ratios and all survive to adulthood without notable spontaneous diseases, which is consistent with a previous report (Zhang et al., 2012). CTL and *Malat1* ^−/−^ littermates showed similar weights for the duration of our experiments between days 50 and 130 (Figure 1B). H&E staining of the CTL and *Malat1* ^−/−^ colonic sections confirmed similar intestine epithelium morphology (Figures 1C, D). To assess intestine barrier function, mice were orally gavaged with 4 kDa Fluorescein isothiocyanate-dextran (FITC-dextran) together with 70 kDa Rhodamine B isothiocyanate-dextran (RITC-dextran). In the CTL and *Malat1* ^−/−^ bloodstream, we found similar levels of RITC-dextran, indicating *Malat1* ^−/−^ mice have intact barrier activity against bacteria-size macromolecules. Interestingly, *Malat1* ^−/−^ mice showed reduced levels of serum FITC-dextran, suggesting that MALAT1 promotes protein-size macromolecule passage in the intestine (Figure 1E).

**FIGURE 2:**
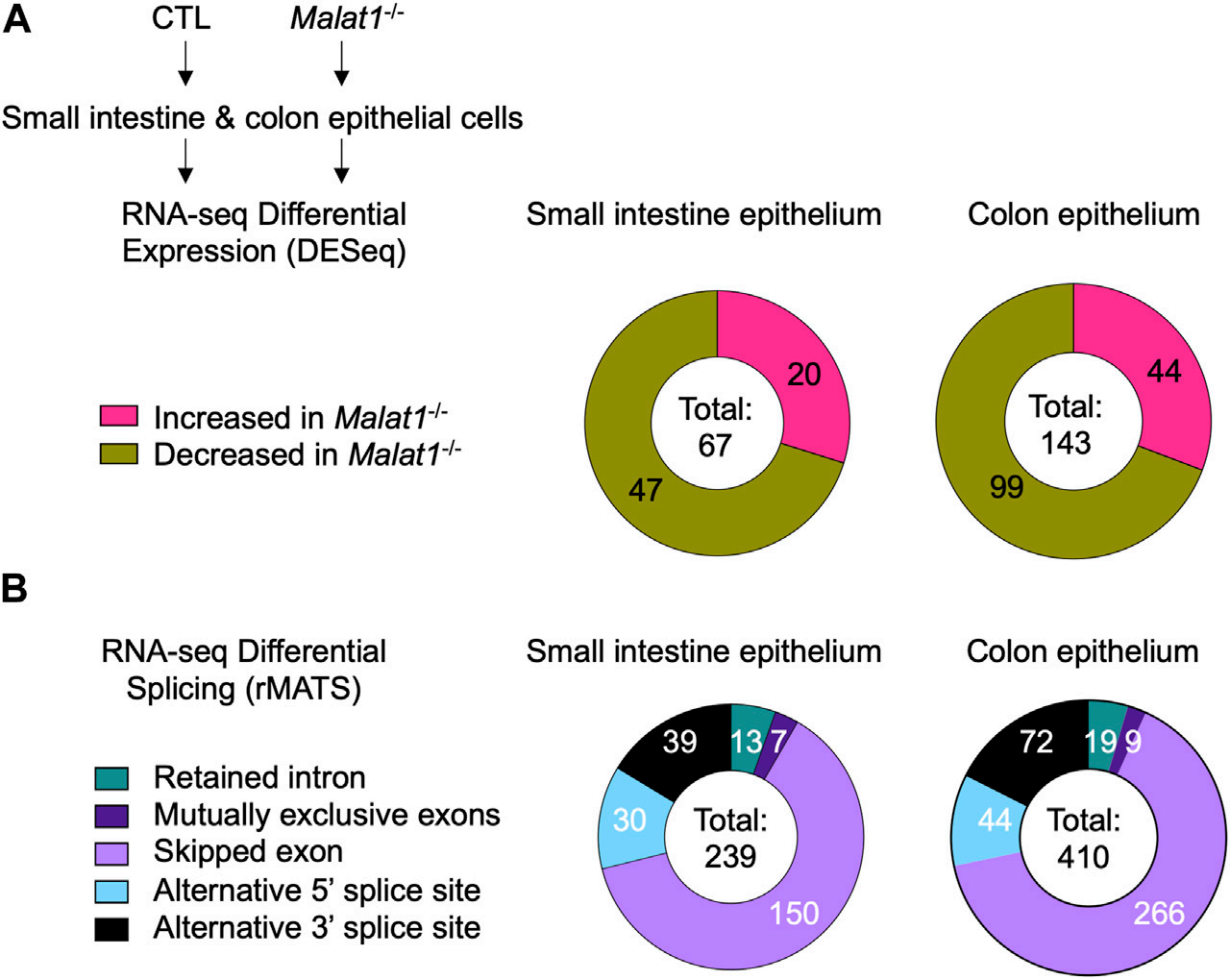
MALAT1 regulates select intestine epithelial cell RNA abundance and splicing. **(A)**. Distribution of MALAT1-dependent genes in the small intestine and colon IECs as determined by DESeq2 in two pairs of cohoused CTL and *Malat1* ^−/−^ littermates (12-week-old females). **(B)**. Distribution of small intestine and colon transcripts relying on MALAT1 for alternative splicing as determined by rMATS in two pairs of cohoused CTL and *Malat1* ^−/−^ littermates (12-week-old females).

To elucidate the mechanism(s) underlying MALAT1 function in the intestine, we isolated small intestine and colon IECs from two pairs of 8-week-old wildtype and MALAT1 knockout female cohoused littermates for differential transcriptome and splicing analyses. Differential gene expression analysis identified 67 and 143 MALAT1-dependent small intestine and colon IEC transcripts, respectively (Figure 2A, Supplementary Table S1). Splicing analysis was performed using rMATS (Shen et al., 2014) and revealed a larger MALAT1-regulatory footprint (Figure 2B, Supplementary Table S2 and Supplementary Table S3). The majority of the MALAT1-dependent alternative splicing events in both small intestine and colon are skipped exons. These results indicate MALAT1 regulates RNA abundance and processing in both the small intestine and colon and that its higher expression levels in the colonic epithelium are associated with a larger set of MALAT1-dependent targets identified in that tissue. We selected two genes that displayed MALAT1-dependent splicing patterns for validation by flow cytometry and confirmed a reduced proportion of TGF-β receptor 1-positive IECs in the *Malat1* ^−/−^ small intestine (Supplementary Figure S1A,B) and an increased proportion of IL-27 receptor alpha-positive IECs in the *Malat1* ^−/−^ colon (Supplementary Figure S1C,D).

**FIGURE 3:**
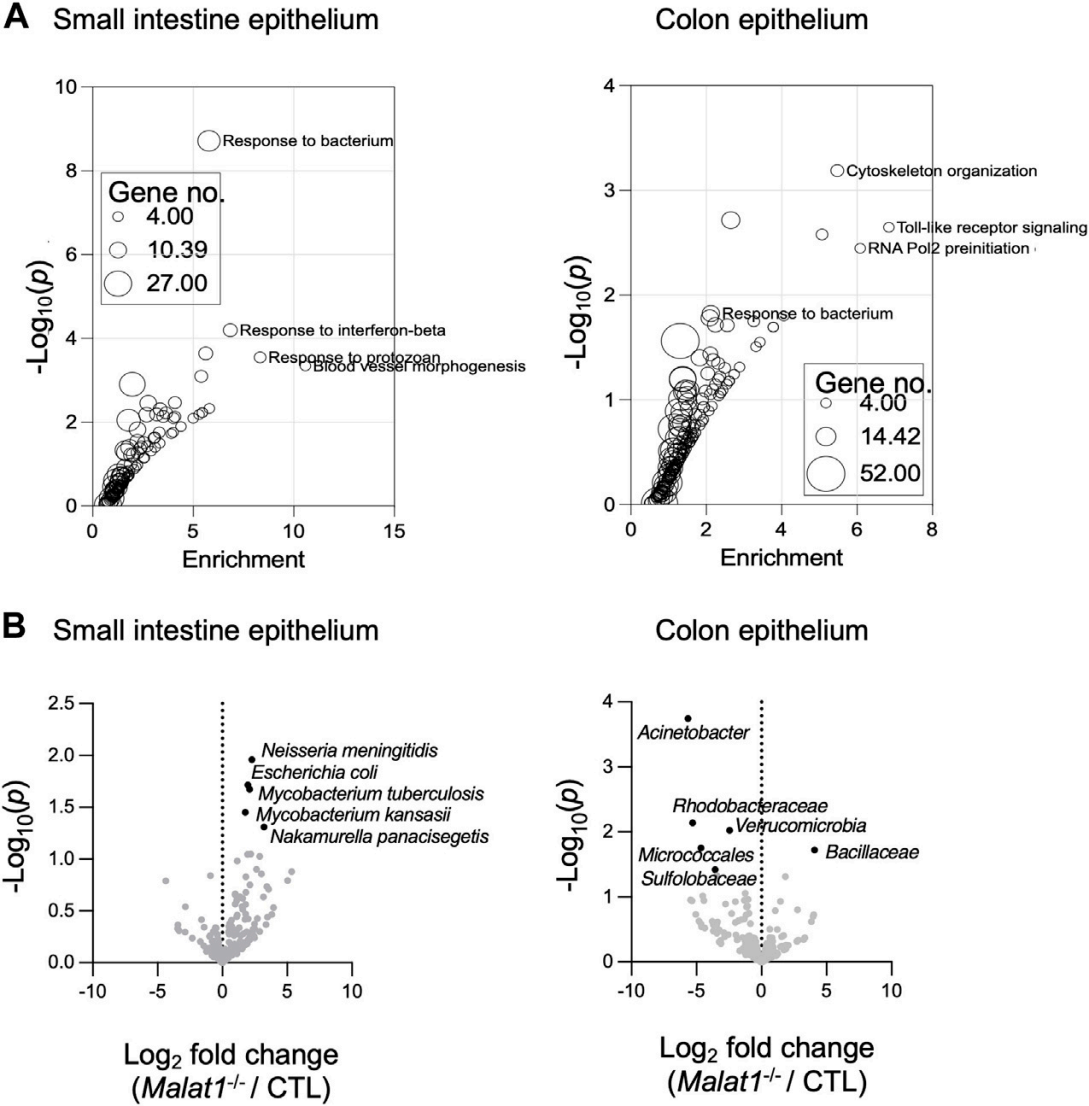
MALAT1-dependent mucosal-associated microbes in the intestine. **(A)**. Gene ontology enrichment analysis of the small intestine and colon epithelium MALAT1 targets from DESeq2 and rMATS combined. The size of the dot is proportional to the number of genes mapping to the enriched GO term. **(B)**. Volcano plots of meta-transcriptomic analysis of small intestine and colon IEC RNA-seq data from two pairs of cohoused CTL and Malat1^−/−^ littermates (12-week-old females).

Gene Ontology analysis of the IEC genes relying on MALAT1 at the expression and/or splicing levels revealed enrichment for pathways implicated in anti-microbial responses in both the small intestine and colon (Figure 3A and Supplementary Figure S2A). Therefore, we performed meta-genomic analysis to test whether alterations in anti-microbial response programs in the *Malat1*-deficient epithelium may be associated with changes to the mucosal-associated microbial communities. Interestingly, we identified an increase of *Neisseria meningitidis*, *Escherichia coli*, *Mycobacterium tuberculosis*, *Mycobacterium kansasii*, and *Nakamurella panacisegetis* in the MALAT1-deficient small intestine (Figure 3B). In the MALAT1-deficient colon, however, there was a decrease of *Acinetobacter*, *Rhodobacteraceae*, *Varucomicrobia*, *Micrococcales* and *Sulfolobaceae*. These results suggest that the impact of MALAT1 on the intestine microbial communities is region-specific.

### MALAT1 negatively regulates polyposis in the small intestine and colon

**FIGURE 4:**
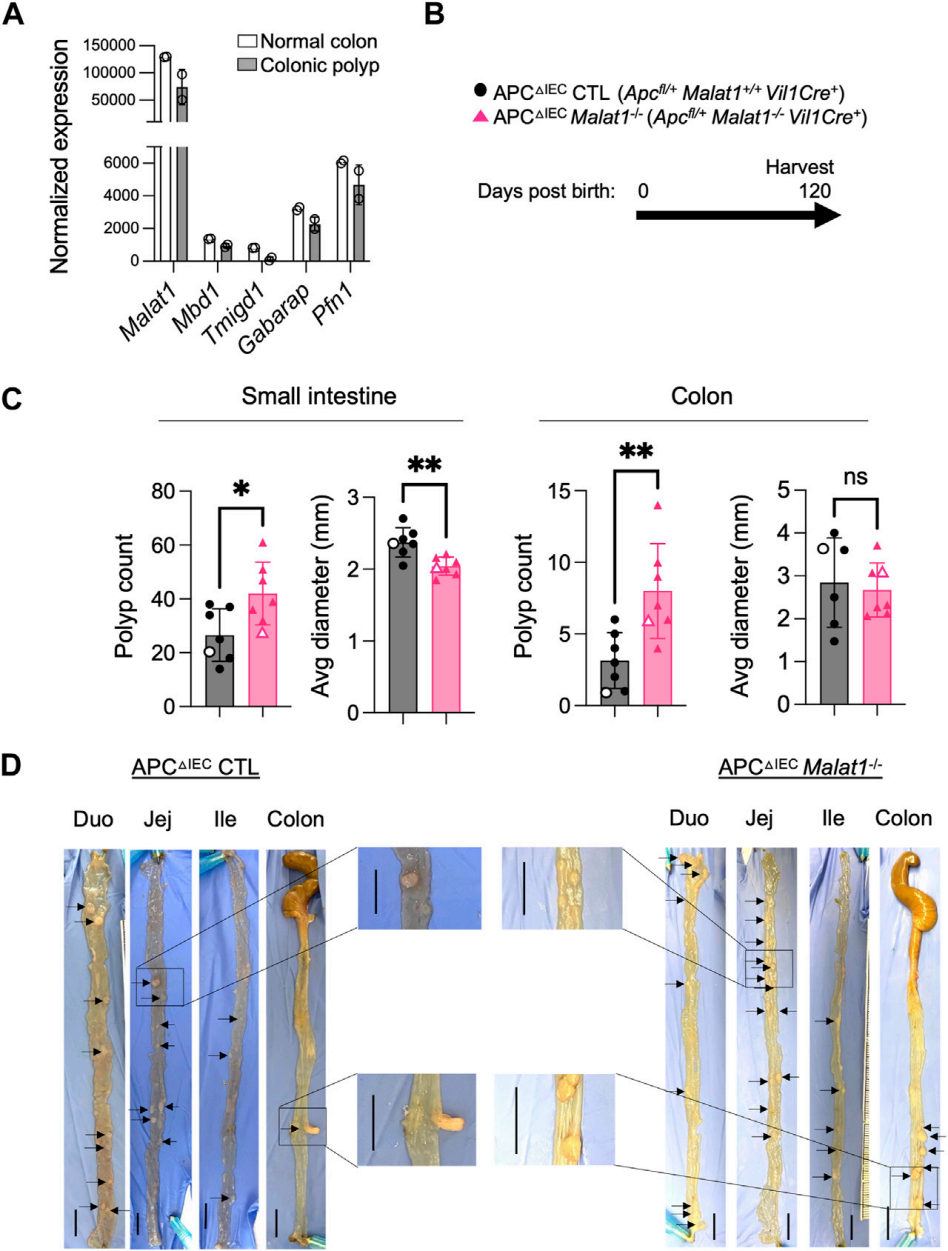
MALAT1 negatively regulates polyposis in the small intestine and colon. **(A)**. Normalized read counts of MALAT1 and select genes encoding known tumor suppressors in colorectal cancer in the steady state colonic epithelium and colonic polyps from the APC^ΔIEC^ mice (GSE146014). **(B)**. Experimental scheme of APC^ΔIEC^ CTL and APC^ΔIEC^ *Malat1* ^−/−^ mice. **(C)**. Polyp count and average polyp diameter in the small intestine (combined counts of the duodenum, jejunum, and ileum) and colon of 120-day old APC^ΔIEC^ CTL (circle, *n* = 7) and APC^ΔIEC^ *Malat1* ^−/−^ mice (triangle, *n* = 7). Each dot represents the results from one mouse. Open circles/triangles indicate the results from the tissues shown in Figure 3D. * *p*-value<0.05, ** *p*-value<0.01, n.s. not significant (*t*-test). **(D)** Representative bright-field images of tumor-bearing small intestine and colons from a pair of APC^△cIEC^ CTL and APC^△cIEC^ *Malat1* ^−/−^ mice from **(C)**. Scale bar equals 1 cm.

Previous studies reported that dysregulated intestine microbiota and altered host anti-microbial responses can modulate individual risks for developing intestine inflammation and cancer (Louis et al., 2014). In human colorectal cancers, MALAT1 transcripts were downregulated relative to normal tissue (Kwok et al., 2018). Compared to primary Stage I lesions, MALAT1 levels were elevated in Stage IV and metastatic lesions (Ji et al., 2019; Zhang et al., 2020). These results suggest that MALAT1 is dynamically regulated during tumorigenesis and may contribute to multiple aspects of colorectal cancer pathogenesis in a stage-specific manner. We then tested whether MALAT1-deficiency influences disease susceptibility in a tumorigenesis setting. To address this question, we employed a mouse model of human familial adenomatous polyposis known as the APC mutant line (Kwong and Dove, 2009). In this model, haploinsufficiency of the tumor suppressor APC results in hyperactivation of WNT and early onset of epithelial dysplasia (Fodde et al., 1994; Hinoi et al., 2007; Kwong and Dove, 2009; Grivennikov et al., 2012; Wang et al., 2014). Similar to human colorectal cancers (Kwok et al., 2018), MALAT1 is slightly downregulated in the murine colonic polyps compared to healthy tissues, a pattern also observed in genes encoding known colorectal cancer tumor suppressor molecules, such as *Mbd1* and *Tmigd1* (Qi and Ding, 2017; Mu et al., 2022) (Figure 4A). To assess the role of MALAT1 in intestine tumorigenesis, we crossed the MALAT1 knockout mice to the *Apc* ^fl/+^ *Vil1*Cre^+^ (APC^ΔIEC^) line (Figure 4B). In the small intestine and colon, APC^ΔIEC^ *Malat1* ^−/−^ mice harbored more polyps than MALAT1-expressing mice (Figures 4C, D). Interestingly, the average sizes of the small intestine polyps in the APC^ΔIEC^ *Malat1* ^−/−^ mice were smaller than those found in the MALAT1-expressing mice. These results revealed a surprising bivalent role of MALAT1 in restricting intestine polyp generation and later promoting aberrant polyp growth.

**FIGURE 5:**
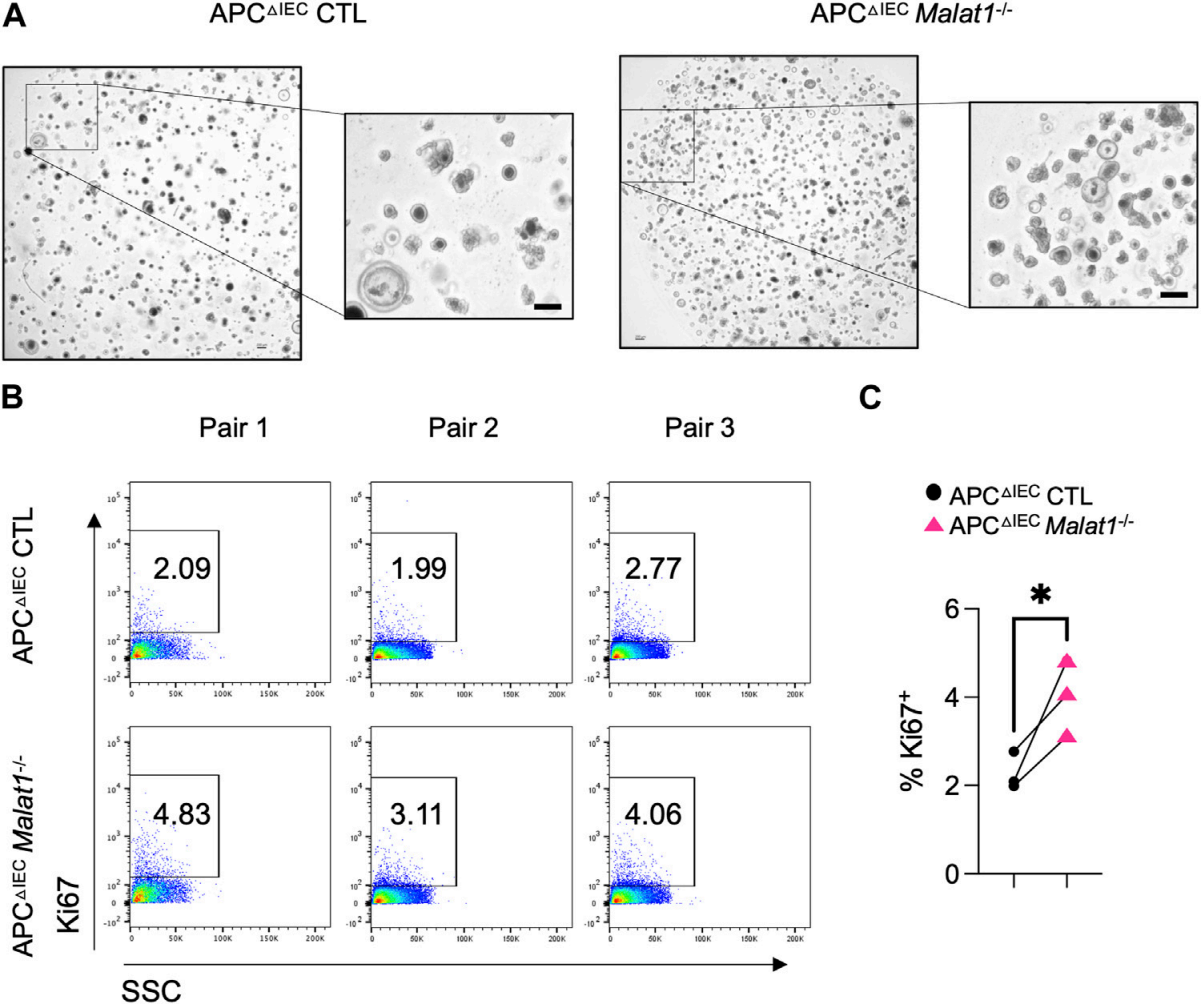
MALAT1 negatively regulates intestine organoid colony formation and proliferation. **(A)**. Representative brightfield images of organoids derived from APC^ΔIEC^ CTL and APC^ΔIEC^ *Malat1* ^−/−^ colonic crypt stem cells. Scale bar represents 200 μm. **(B)**. Representative flow cytometry analysis of intracellular Ki67 staining in live APC^ΔIEC^ CTL and APC^ΔIEC^ *Malat1* ^−/−^ colonic organoid cells (Live Epcam^+^). SSC: side scatter as an index for cell granularity. **(C)**. The proportion of Ki67^+^ colonic organoid cells in the APC^ΔIEC^ CTL and APC^ΔIEC^ *Malat1* ^−/−^ cultures. Each dot represents the result from one independent experiment. * *p*-value<0.05 (*t*-test).

RNAs from intestine polyps and adjacent normal tissues were harvested and assessed for the expression of *Ctnnb1* encoding β-Catenin and *Ki67* as an index of cell proliferation. In the small intestine polyps and adjacent normal tissues, *Ctnnb1* and *Ki67* levels negatively correlate with the *Malat1* gene dosage (Supplementary Figure S3 and Supplementary Figure S4). In contrast, expression of the tumor stem cell marker *Cd44* was MALAT1-independent. To determine the epithelial cell-intrinsic role of MALAT1 in polyposis, we purified and cultured colonic crypt stem cells from APC^ΔIEC^ CTL and APC^ΔIEC^ *Malat1* ^−/−^ mice and assessed their capacity to establish colonies on Matrigel *in vitro*. Overall, APC^ΔIEC^ *Malat1* ^−/−^ colonies were more abundant than those derived from APC^ΔIEC^ CTL cells (Figure 5A). Flow cytometry analysis revealed that the APC^ΔIEC^ *Malat1* ^−/−^ cultures harbored a larger fraction of Ki67-positive actively proliferating population (Figures 5B, C). These results suggest that MALAT1 in epithelial cells is a negative regulator of colony establishment *in vitro*.

### MALAT1 occupies the chromatin regulatory elements on a subset of its epithelial target genes

**FIGURE 6:**
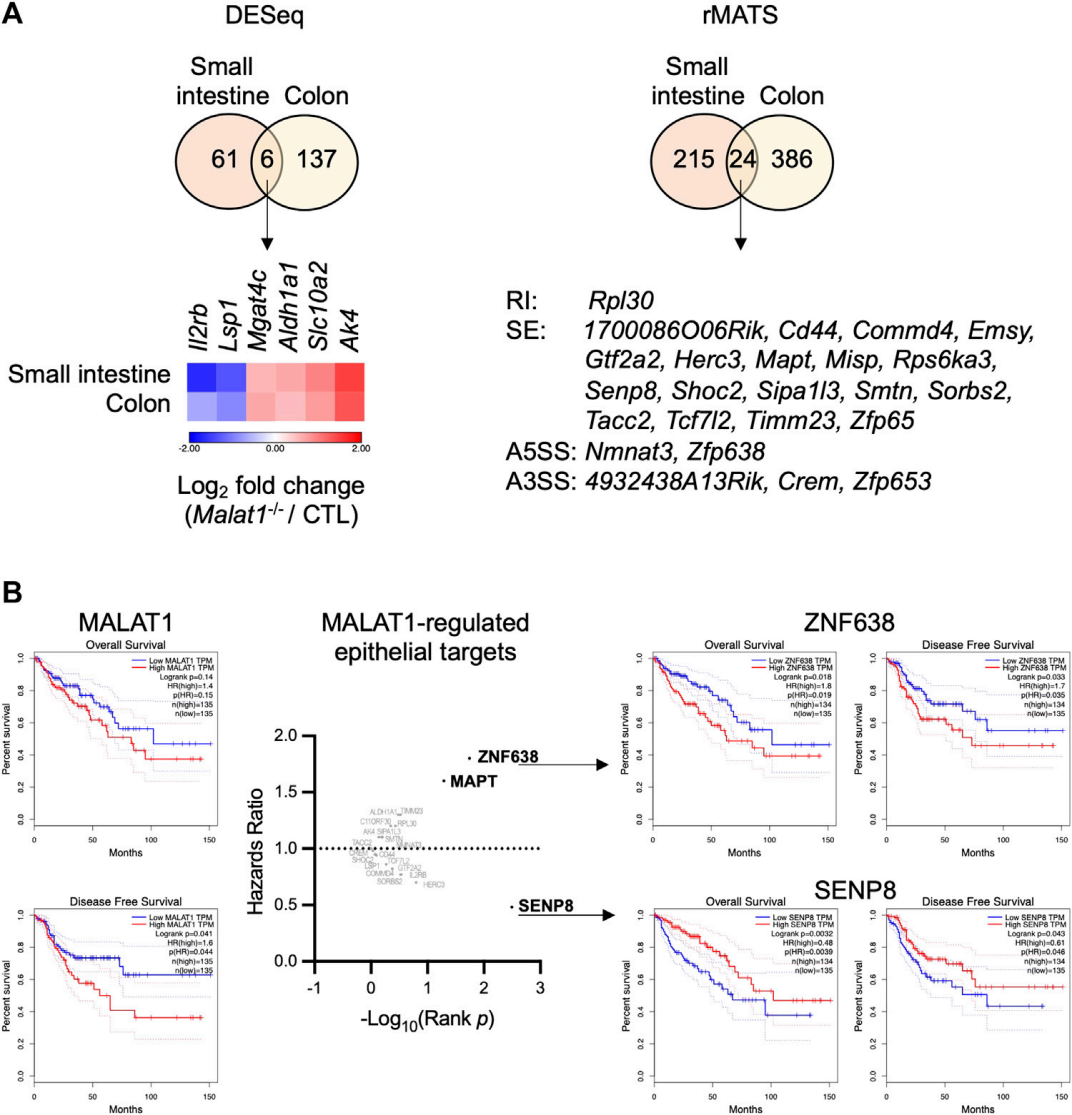
Select MALAT1-dependent genes in predicting colorectal cancer patient hazard risks. **(A)**. Overlap analysis of the differential expressed genes and MALAT1-dependent splicing events in the small intestine and colon. RI: retained intron. SE: skipped exon. A5SS: alternative 5′ splice site. A3SS: alternative 3′ splice site. **(B)**. Overall survival and disease-free survival among colon adenocarcinoma patients with high or low expression of each MALAT1 target identified in **(A)**.

We hypothesized that MALAT1 suppresses polyposis in the small intestine and colon by regulating a common set of targets in both intestine regions that are involved in epithelial cell transformation. To identify these targets, we overlaid the small intestine and colon MALAT1-dependent transcripts identified from the DESeq and rMATS analysis and found 30 targets that were dependent on MALAT1 at the overall RNA expression or alternative splicing levels (Figure 6A), including those encoding an acetylglucosaminyltransferase MGAT4C, an aldehyde dehydrogenase ALDH1A1, and an adenylate kinase AK4 that have been previously implicated in other types of cancers. In addition, two of the MALAT1 targets shared across the small intestine and colon, ZNF638 and SENP8, are associated with a change in hazards ratio for overall survival and disease-free survival in human colon adenocarcinoma patients (Figure 6B). These results suggest that MALAT1 downstream targets may contribute to cancer pathogenesis in humans.

**FIGURE 7:**
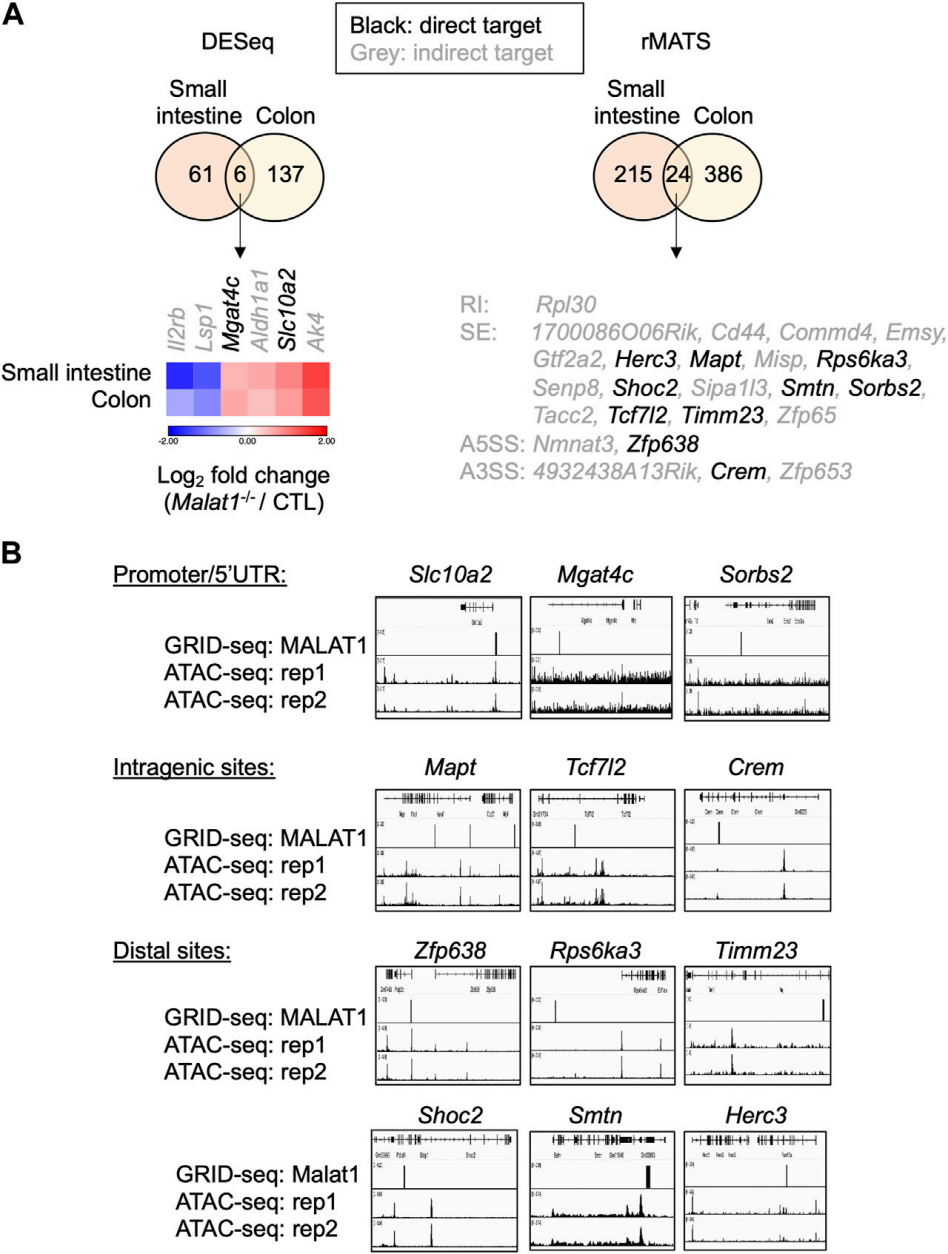
MALAT1 occupies the chromatin regulatory elements on a subset of its epithelial target genes. **(A)**. Venn diagrams of overlapping differential expressed genes and MALAT1-dependent splicing events between small and large intestine. Black: MALAT1 chromatin occupancy detected at or near the gene body. Grey: MALAT1 not recruited to nearby chromatin. **(B)**. IGV browser views of MALAT1 GRID-seq and open chromatin (ATAC-seq) signals on select MALAT1-direct target genes from A (Tang et al., 2017). Rep, biological replicate.

To determine whether these targets were regulated by MALAT1 at the chromatin level and/or through more complex mechanisms, we employed the GRID-seq assay to characterize the chromatin occupancy of MALAT1 in small intestine epithelium as previously described (Li et al., 2017b; Zhou et al., 2019). In the small intestine epithelium, 12 of the 30 MALAT1 target genes identified earlier had MALAT1 binding near the gene locus (Figure 7A). For example, MALAT1 occupied the promoter/5′UTR region on *Mgat4c*, *Slc10a2,* and *Sorbs2*, intragenic regions of *Mapt*, *Tcf7l2*, and *Crem*, and distal elements on *Zfp638*, *Rps6ka3*, *Timm23*, *Shoc2*, *Smtn*, and *Herc3* (Figure 7B). Chromatin accessibility ATAC-seq assays further indicate that MALAT1 occupied regions in the small intestine epithelium lied within both regions of open and closed chromatin in a gene-specific manner. These results suggest that MALAT1 regulates intestine epithelial cell gene programs through both direct and indirect mechanisms.

## Discussion

MALAT1 is dysregulated in various cancer types, including colorectal cancer (Wei and Niu, 2015; Li et al., 2017a; Arun et al., 2020). Subsequent studies reported that the expression of MALAT1 is an independent prognostic factor and is involved in tumor cell proliferation, metastasis, and epithelial-mesenchymal transition (Ji et al., 2014; Sun et al., 2019). Using the APC mutant mouse model of intestine tumorigenesis, our study provides the first *in vivo* evidence that MALAT1 restricts polyp formation in both the small intestine and colon. Knocking out MALAT1 results in the generation of a larger number of intestine polyps. In addition to a role in restricting polyp formation, MALAT1 also contributes to abnormal intestinal polyp growth at a later stage. The polyps found in the MALAT1 knockout small intestine were smaller in size than those found in MALAT1-expressing mice. More detailed histological analysis in the future will be needed to determine the exact mechanism underlying MALAT1’s bivalent roles in polyp initiation and growth at different disease stages.

We speculate that MALAT1 protects against polyp formation by regulating a set of target genes involved in tumorigenesis shared by both the small intestine and colon. Among the thirty MALAT1 targets commonly found in the small intestine and colon, many have been previously implicated in other types of cancers. Most interestingly, we identified two MALAT1-dependent novel molecules, ZNF638 and SENP8, that are associated with significant alterations in colon adenocarcinoma patients’ overall survival and disease-free survival. One limitation of our current transcriptomic analysis is the reliance on two pairs of control and MALAT1-deficient mice. While we have validated a few of the MALAT1-dependent IEC targets using other approaches such as flow cytometry on independent sets of mice, future transcriptomic studies powered by a larger experimental cohort will be needed to further validate these initial findings.

MALAT1 has been implicated in immune regulation, including modulating pro-inflammatory cytokine production and activating T cells and natural killer cells upon bacterial or viral infections (Kakaradov et al., 2017; Kim et al., 2017; Kanbar et al., 2022). Our findings now provide *in vivo* evidence that MALAT1 regulates anti-microbial responses in intestine epithelial cells at the levels of gene expression and alternative splicing and influences intestinal microbiome composition. Our mechanistic studies further identified MALAT1-occupied chromatin sites in the intestine epithelium genome-wide. Coupled with our transcriptomic studies, these results suggest that MALAT1 regulates intestinal epithelial gene programs by direct binding to target gene chromatin and/or indirect mechanisms. Collectively, these findings provide new insights into the function of MALAT1 in the intestine epithelium and its potential as a prognostic marker as well as a therapeutic target in intestinal diseases.

## Materials and methods

### Mice

C57BL/6 wild-type were obtained from the Jackson Laboratory. Malat1^−/−^ mice were obtained from Dr. David Spector’s laboratory and have been previously described (Zhang et al., 2012). Heterozygous mice were bred to yield 8–12-week old cohoused littermates for transcriptomic and genomic studies. *Apc* ^flox^ mice were obtained from Dr. Eric Fearon’s Laboratory and previously described in reference (Grivennikov et al., 2012). Intestine tissues were harvested from 120 to 130-day-old CTL and *Malat1* ^−/−^ in the *Apc* ^*f*lox^ background to assess tumor burden. Tumor measurements were determined by double-blinded analyses using ImageJ. Both male and female mice were used in the experiments described. All animal studies were approved and followed the Institutional Animal Care and Use Guidelines of the University of California San Diego (Protocol #S16156). Our vivarium at the University of California San Diego is kept under specific pathogen-free conditions. Regular serology and PCR tests are used to monitor and ensure the absence of epizootic diarrhea of infant mouse virus (EDIM), mouse hepatitis virus (MHV), mouse parvovirus (MPV), minute virus of mice (MVM), Theiler’s murine encephalomyelitis virus (TMEV), fur mites and pinworms. Colonic sections from 8-week-old littermates were stained with H&E and scored for changes in the inflammatory infiltrate, submucosal inflammation, crypt morphology, and muscle thickening in a double-blind fashion as described in (Abbasi et al., 2020).

### Intestinal permeability assay

Mice were deprived of food and bedding for 4 h prior to oral gavage with 4 kDa Fluorescein isothiocyanate–dextran (Millipore-Sigma, FD4, 100 mg/kg) together with 70 kDa Rhodamine B isothiocyanate-dextran (Millipore-Sigma, R9379, 50 mg/kg). Blood samples were taken 4 h post-gavage by submandibular bleed. The FD4 and Rhodamine signals were measured using a TECAN fluorescent plate reader at the excitation/emission wavelengths of 485/535 and 540 nm/585 nm, respectively.

### Intestinal crypt isolation and organoid culture

Colonic crypts were isolated according to the manufacturer’s recommendation (STEMCELL, technical bulletin #28223). Briefly, intestine tissues were harvested from 6–8-week-old CTL and *Malat1* ^−/−^ in the *Apc* ^flox^ background and cut into 2 mm pieces. After 20 washes in cold PBS, tissues were resuspended in 25 mL room temperature Gentle Cell Dissociation Reagent (STEMCELL, #07174) and incubated at room temperature for 15 min on a rocking platform at 20 rpm. The pellets enriched with intestinal crypts were resuspended in cold PBS containing 0.1% BSA. Isolated colonic crypts were embedded in Corning^®^ Matrigel^®^ Matrix (Corning™ 356231) and seeded onto pre-warmed, non-treated 24-well plates (CytoOne^®^ by StarLab) and overlaid with conditioned media (STEMCELL, #6005) as described previously (Miyoshi and Stappenbeck, 2013). Organoid pictures were imaged using a Keyence bz-x800 microscope at ×20 magnification with image stacks capturing the entire organoid volume.

### Flow cytometry analysis

Intestinal epithelial cells were surface stained with LIVE/DEAD Fixable Cell stain (ThermoFisher, L34957), and fluorescent conjugated antibodies against EpCAM, TGFBR1, and IL27RA (see Supplementary Table S4 for detailed information, 1:400 in PBS) for 30 min. For intracellular staining of Ki67, cells were first fixed/permeabilized (ThermoFisher Cat: 00-5521-00) and then incubated with the anti-Ki67 antibody for 1 h at room temperature. Intestine epithelial cells were defined as live Epcam^+^. Flow cytometry data was analyzed with FlowJo (version 10.8.1).

### cDNA synthesis and qPCR

Total RNA was extracted with the RNeasy Plus kit (QIAGEN) and reverse transcribed using iScript™ Select cDNA Synthesis Kit (Bio-Rad). Real time RT-PCR was performed using iTaq™ Universal SYBR^®^ Green Supermix (Bio-Rad). Results were normalized to mouse *Hprt*. Primers were designed using Primer-BLAST to span across splice junctions, resulting in PCR amplicons that span at least one intron. Primer sequences are listed in Supplementary Table S4.

### RNA-seq analysis

Small intestine and colonic epithelial cells from two pairs of *Malat1* ^−/−^ and CTL cohoused littermates were enriched as previously described in (Abbasi et al., 2020). Ribosome-depleted RNAs were used to prepare sequencing libraries. 100 bp paired-end sequencing was performed on an Illumina HiSeq4000 by the Institute of Genomic Medicine (IGM) at the University of California San Diego. Each sample yielded approximately 30–40 million reads. Paired-end reads were aligned to the mouse mm10 genome with the STAR aligner version 2.6.1a (Dobin et al., 2013) using the parameters: “--outFilterMultimapNmax 20 --alignSJoverhangMin 8 --alignSJDBoverhangMin 1 --outFilterMismatchNmax 999 --outFilterMismatchNoverReadLmax 0.04 --alignIntronMin 20 --alignIntronMax 1000000 --alignMatesGapMax 1000000”. Uniquely mapped reads overlapping with exons were counted using featureCounts (Liao et al., 2014) for each gene in the GENCODE. vM19 annotation. Differential expression analysis was performed using DESeq2 (v1.18.1 package) (Love et al., 2014), including a covariate in the design matrix to account for differences in harvest batch/time points. Regularized logarithm (rlog) transformation of the read counts of each gene was carried out using DESeq2. Pathway analysis was performed on differentially expressed protein coding genes with minimal counts of 10, log_2_ fold change cutoffs of ≥0.5 or ≤ −0.5, and *p*-values < 0.05 using Gene Ontology (http://www.geneontology.org/) where all expressed genes in the specific cell type were set as background.

Alternative splicing events were analyzed by Multivariate analysis of transcript splicing (rMATS (Shen et al., 2014)) using the parameters “python rmats. py --b1/path/to/b1. txt --b2/path/to/b2. txt–gtf/path/to/the.gtf -t paired --readLength 150 --nthread 4 --od/path/to/output --tmp/path/to/tmp_output”. Using a 0.01 FDR cutoff, MALAT1-dependent splicing events were identified.

For metatranscriptomic analysis of ileal associated microbial populations, reads from the CTL and Malat1^−/−^ IEC RNA-seq dataset that were not mapped to the mouse genome were assigned with taxonomic labels using Kraken V.1. The standard Kraken database encompassing annotated bacterial, archaeal, and viral genomes was used for classification of sequences with the command: “kraken --classified-out/path/to/classified_fq --unclassified-out/path/to/unclassified. fq --db $DBNAME --paired --fastq-input pair1. fa pair2. fa >/path/to/results”. A Kraken report was generated with the command: “kraken-report --$DBNAME kraken. output” (Wood and Salzberg, 2014). Differential microbial counts were assessed by DEseq2 cut-off of *p* < 0.05 with the Wald test and Log2 fold change (*Malat1* ^−/−^/CTL) > 1.5 or ≤1.5.

### GRID-seq

GRID-seq of small intestine epithelial cells were performed as described (Li et al., 2017b; Zhou et al., 2019). Briefly, two independent biological replicates (5–10 × 10^6^) were crosslinked with disuccinimidyl glutarate (DSG) and formaldehyde. DNA in isolated nuclei were digested with AluI. A biotinylated bivalent linker was ligated to chromatin-associated RNA (with the ssRNA stretch on the linker) and nearby fragmented genomic DNA and captured by streptavidin microbeads for library construction. Single-end sequencing was performed on HiSeq400 (Illumina, 200 million reads/sample). Raw sequencing reads were evaluated by FastQC (Gu et al., 2014). Reads below 85bp were filtered out and those above 90bp with high quality were trimmed by Cutadapt (Martin, 2011) as suggested according to previous report (Li et al., 2017b; Zhou et al., 2019). The GRID-seq linker position at each read and the paired reads originated from RNA or genomic DNA were identified by matefq in GridTools (Zhou et al., 2019). Paired RNA and DNA reads were then mapped to the mouse genome (GRCm38/mm10) by BWA respectively. Uniquely paired reads were used to generate a set of RNA-DNA interaction matrix for downstream analyses in the GridTools pipeline. Read counts from the two repeats were summarized into two 1 kb genomic bins. Chromatin enriched with MALAT1 RNAs (GRID-seq peak call) were defined as 2 kb regions with clustered MALAT1 signals above the background signal expected from random interactions (>5-fold changes).

### ATAC-seq

ATAC-seq libraries were generated as described in (Buenrostro et al., 2015). ATAC-seq processing followed the ENCODE guideline with some modifications (Landt et al., 2012). Specifically, single-end raw reads were mapped to the mouse genome (GENCODE assembly mm10) by bowtie2 (Version 2.3.4.1) in the local mapping mode with parameter “--local”, followed by PCR deduplication by SAMTools (Version 1.9) with the utility markedup (Li et al., 2009). Mapped reads from each sample repeats were merged into a single BAM file by SAMTools, and peaks were called using MACS2 (Version 2.2.6) (Zhang et al., 2008) with “callpeak --nomodel --extsize 100”. Regions with peak-score below 30 were filtered out and the remaining reliable peak profiles were transformed into bigwig format and visualized on the Integrative Genomics Viewer (IGV Version 2.8.2) (Thorvaldsdottir et al., 2013).

### Statistical analysis

The values are presented as the mean ± standard deviation (SD). Statistical significance was evaluated using GraphPad Prism V.8 software (GraphPad). The *t*-test was used to determine significant differences between groups. A *p*-value of less than 0.05 was considered statistically significant in all experiments.

## Data Availability

The datasets presented in this study can be found in online repositories. The names of the repository/repositories and accession number(s) can be found below: https://www.ncbi.nlm.nih.gov/geo/ under accession numbers: GSE208232, GSE226199, and GSE210186.

## Supplementary material

SUPPLEMENTARY FIGURE S1 MALAT1-dependent target validation. **(A)** IGV browser views of CTL (black) and *Malat1* ^−/−^ (grey) RNA-seq signals at the *Tgfbr1* locus. Connecting lines indicate reads spanning exon junctions. **(B)** The proportion of TGFBR1-expressing small intestine epithelial cells (Epcam^+^) in three pairs of 12-week-old CTL and Malat1 littermates as determined by flow cytometry. Each dot represents the result from one mouse. * *p*-value < 0.05 (*t*-test). **(C)** IGV browser views of CTL (black) and Malat1 (grey) RNA-seq signals at the Il27ra locus. Connecting lines indicate reads spanning exon junctions. **(D)** The proportion of IL-27RA-expressing colonic epithelial cells (Epcam+) from two pairs of 12-week-old CTL and Malat1 littermates as determined by flow cytometry. Each dot represents the result from one mouse. * *p*-value < 0.05 (*t*-test).

SUPPLEMENTARY FIGURE S2 MALAT1-dependent genes involved in response to bacteria. **(A)** MALAT1-dependent gene network in the small intestine and colonic epithelium. Each orange dot represents one gene and blue nodes indicate pathway annotation. The color of the nodes represents the number of MALAT1-dependent genes associated with the specific annotation term and the size of the node represents the -log_10_(adjusted p-value).

SUPPLEMENTARY FIGURE S3 Select small intestine gene expression in the polyposis model. **(A)** *Ctnnb1*, *Ki67*, and *Cd44* mRNA expression in ileal polyps and adjacent normal tissues from APC^ΔIEC^ *Malat1* ^+/+^ (*n* = 3), *Malat1* ^+/−^ (*n* = 5), and *Malat1* ^−/−^ (*n* = 7) mice harvested on day 120. Levels were normalized to housekeeping gene *Hprt.* Adj Norm, adjacent normal tissue. *, *p*-value < 0.05, n.s. not significant (Welch’s *t*-test).

SUPPLEMENTARY FIGURE S4 Select colonic gene expression in the polyposis model. **(A)** *Ctnnb1*, *Ki67*, and *Cd44* mRNA expression in colonic polyps and adjacent normal tissues from APC^ΔIEC^ *Malat1* ^+/+^ (*n* = 2), *Malat1* ^+/−^ (*n* = 3), and *Malat1* ^−/−^ (*n* = 5) mice harvested on day 120. Levels were normalized to housekeeping gene *Hprt.* Adj Norm, adjacent normal tissue. n.s. not significant (Welch’s *t*-test).

## References

[B1] Abbasi N. Long T. Li Y. Yee B. A. Cho B. S. Hernandez J. E. (2020). DDX5 promotes oncogene C3 and FABP1 expressions and drives intestinal inflammation and tumorigenesis. Life Sci. Alliance 3, e202000772. 10.26508/lsa.202000772 32817263 PMC7441524

[B2] Ali A. Tan H. Kaiko G. E. (2020). Role of the intestinal epithelium and its interaction with the microbiota in food allergy. Front. Immunol. 11, 604054. 10.3389/fimmu.2020.604054 33365031 PMC7750388

[B3] Arun G. Aggarwal D. Spector D. L. (2020). MALAT1 long non-coding RNA: Functional implications. Non-Coding RNA 6, 22. 10.3390/ncrna6020022 32503170 PMC7344863

[B4] Buenrostro J. D. Wu B. Chang H. Y. Greenleaf W. J. (2015). ATAC-seq: A method for assaying chromatin accessibility genome-wide. Curr. Protoc. Mol. Biol. 109, 21.29.1–21.29.9. 10.1002/0471142727.mb2129s109 PMC4374986 25559105

[B5] Chang S. M. Hu W. W. (2018). Long non-coding RNA MALAT1 promotes oral squamous cell carcinoma development via microRNA-125b/STAT3 axis. J. Cell. physiology 233, 3384–3396. 10.1002/jcp.26185 28926115

[B6] Chou J. Wang B. Zheng T. Li X. Zheng L. Hu J. (2016). MALAT1 induced migration and invasion of human breast cancer cells by competitively binding miR-1 with cdc42. Biochem. biophysical Res. Commun. 472, 262–269. 10.1016/j.bbrc.2016.02.102 26926567

[B7] Dobin A. Davis C. A. Schlesinger F. Drenkow J. Zaleski C. Jha S. (2013). Star: Ultrafast universal RNA-seq aligner. Bioinformatics 29, 15–21. 10.1093/bioinformatics/bts635 23104886 PMC3530905

[B8] Engreitz J. M. Sirokman K. McDonel P. Shishkin A. A. Surka C. Russell P. (2014). RNA-RNA interactions enable specific targeting of noncoding RNAs to nascent Pre-mRNAs and chromatin sites. Cell 159, 188–199. 10.1016/j.cell.2014.08.018 25259926 PMC4177037

[B9] Fodde R. Edelmann W. Yang K. van Leeuwen C. Carlson C. Renault B. (1994). A targeted chain-termination mutation in the mouse Apc gene results in multiple intestinal tumors. Proc. Natl. Acad. Sci. U. S. A. 91, 8969–8973. 10.1073/pnas.91.19.8969 8090754 PMC44728

[B10] Gong W. J. Yin J. Y. Li X. P. Fang C. Xiao D. Zhang W. (2016). Association of well-characterized lung cancer lncRNA polymorphisms with lung cancer susceptibility and platinum-based chemotherapy response. Tumour Biol. 37, 8349–8358. 10.1007/s13277-015-4497-5 26729200

[B11] Grivennikov S. I. Wang K. Mucida D. Stewart C. A. Schnabl B. Jauch D. (2012). Adenoma-linked barrier defects and microbial products drive IL-23/IL-17-mediated tumour growth. Nature 491, 254–258. 10.1038/nature11465 23034650 PMC3601659

[B12] Gu Z. Gu L. Eils R. Schlesner M. Brors B. (2014). Circlize Implements and enhances circular visualization in R. Bioinformatics 30, 2811–2812. 10.1093/bioinformatics/btu393 24930139

[B13] Guo F. Li Y. Liu Y. Wang J. Li Y. Li G. (2010). Inhibition of metastasis-associated lung adenocarcinoma transcript 1 in CaSki human cervical cancer cells suppresses cell proliferation and invasion. Acta biochimica biophysica Sinica 42, 224–229. 10.1093/abbs/gmq008 20213048

[B14] Hinoi T. Akyol A. Theisen B. K. Ferguson D. O. Greenson J. K. Williams B. O. (2007). Mouse model of colonic adenoma-carcinoma progression based on somatic Apc inactivation. Cancer Res. 67, 9721–9730. 10.1158/0008-5472.CAN-07-2735 17942902

[B15] Ji P. Diederichs S. Wang W. Böing S. Metzger R. Schneider P. M. (2003). MALAT-1, a novel noncoding RNA, and thymosin beta4 predict metastasis and survival in early-stage non-small cell lung cancer. Oncogene 22, 8031–8041. 10.1038/sj.onc.1206928 12970751

[B16] Ji Q. Cai G. Liu X. Zhang Y. Wang Y. Zhou L. (2019). MALAT1 regulates the transcriptional and translational levels of proto-oncogene RUNX2 in colorectal cancer metastasis. Cell Death Dis. 10, 378. 10.1038/s41419-019-1598-x 31097689 PMC6522477

[B17] Ji Q. Zhang L. Liu X. Zhou L. Wang W. Han Z. (2014). Long non-coding RNA MALAT1 promotes tumour growth and metastasis in colorectal cancer through binding to SFPQ and releasing oncogene PTBP2 from SFPQ/PTBP2 complex. Br. J. Cancer 111, 736–748. 10.1038/bjc.2014.383 25025966 PMC4134507

[B18] Kakaradov B. Arsenio J. Widjaja C. E. He Z. Aigner S. Metz P. J. (2017). Early transcriptional and epigenetic regulation of CD8+ T cell differentiation revealed by single-cell RNA sequencing. Nat. Immunol. 18, 422–432. 10.1038/ni.3688 28218746 PMC5360497

[B19] Kanbar J. N. Ma S. Kim E. S. Kurd N. S. Tsai M. S. Tysl T. (2022). The long noncoding RNA Malat1 regulates CD8+ T cell differentiation by mediating epigenetic repression. J. Exp. Med. 219, e20211756. 10.1084/jem.20211756 35593887 PMC9127983

[B20] Kim S. H. Kim S. H. Yang W. I. Kim S. J. Yoon S. O. (2017). Association of the long non-coding RNA MALAT1 with the polycomb repressive complex pathway in T and NK cell lymphoma. Oncotarget 8, 31305–31317. 10.18632/oncotarget.15453 28412742 PMC5458209

[B21] Kwok Z. H. Roche V. Chew X. H. Fadieieva A. Tay Y. (2018). A non-canonical tumor suppressive role for the long non-coding RNA MALAT1 in colon and breast cancers. Int. J. Cancer 143, 668–678. 10.1002/ijc.31386 29574704

[B22] Kwong L. N. Dove W. F. (2009). APC and its modifiers in colon cancer. Adv. Exp. Med. Biol. 656, 85–106. 10.1007/978-1-4419-1145-2_8 19928355 PMC3754875

[B23] Landt S. G. Marinov G. K. Kundaje A. Kheradpour P. Pauli F. Batzoglou S. (2012). ChIP-seq guidelines and practices of the ENCODE and modENCODE consortia. Genome Res. 22, 1813–1831. 10.1101/gr.136184.111 22955991 PMC3431496

[B24] Li H. Handsaker B. Wysoker A. Fennell T. Ruan J. Homer N. (2009). The sequence alignment/map format and SAMtools. Bioinformatics 25, 2078–2079. 10.1093/bioinformatics/btp352 19505943 PMC2723002

[B25] Li P. Zhang X. Wang H. Wang L. Liu T. Du L. (2017). MALAT1 is associated with poor response to oxaliplatin-based chemotherapy in colorectal cancer patients and promotes chemoresistance through EZH2. Mol. cancer Ther. 16, 739–751. 10.1158/1535-7163.Mct-16-0591 28069878

[B26] Li Q. Dai Y. Wang F. Hou S. (2016). Differentially expressed long non-coding RNAs and the prognostic potential in colorectal cancer. Neoplasma 63, 977–983. 10.4149/neo_2016_617 27596298

[B27] Li X. Zhou B. Chen L. Gou L. T. Li H. Fu X. D. (2017). GRID-seq reveals the global RNA-chromatin interactome. Nat. Biotechnol. 35, 940–950. 10.1038/nbt.3968 28922346 PMC5953555

[B28] Li Y. Zhu L. Chen P. Wang Y. Yang G. Zhou G. (2021). MALAT1 maintains the intestinal mucosal homeostasis in crohn’s disease via the miR-146b-5p-CLDN11/NUMB pathway. J. Crohn's Colitis 15, 1542–1557. 10.1093/ecco-jcc/jjab040 33677577

[B29] Liao Y. Smyth G. K. Shi W. (2014). featureCounts: an efficient general purpose program for assigning sequence reads to genomic features. Bioinformatics 30, 923–930. 10.1093/bioinformatics/btt656 24227677

[B30] Louis P. Hold G. L. Flint H. J. (2014). The gut microbiota, bacterial metabolites and colorectal cancer. Nat. Rev. Microbiol. 12, 661–672. 10.1038/nrmicro3344 25198138

[B31] Love M. I. Huber W. Anders S. (2014). Moderated estimation of fold change and dispersion for RNA-seq data with DESeq2. Genome Biol. 15, 550. 10.1186/s13059-014-0550-8 25516281 PMC4302049

[B32] Luan W. Li L. Shi Y. Bu X. Xia Y. Wang J. (2016). Long non-coding RNA MALAT1 acts as a competing endogenous RNA to promote malignant melanoma growth and metastasis by sponging miR-22. Oncotarget 7, 63901–63912. 10.18632/oncotarget.11564 27564100 PMC5325412

[B33] Martin M. (2011). Cutadapt removes adapter sequences from high-throughput sequencing reads. EMBnet. J. 17, 10. 10.14806/ej.17.1.200

[B34] Miyagawa R. Tano K. Mizuno R. Nakamura Y. Ijiri K. Rakwal R. (2012). Identification of cis- and trans-acting factors involved in the localization of MALAT-1 noncoding RNA to nuclear speckles. RNA (New York, N.Y.) 18, 738–751. 10.1261/rna.028639.111 22355166 PMC3312561

[B35] Miyoshi H. Stappenbeck T. S. (2013). *In vitro* expansion and genetic modification of gastrointestinal stem cells in spheroid culture. Nat. Protoc. 8, 2471–2482. 10.1038/nprot.2013.153 24232249 PMC3969856

[B36] Mu L. Wang Y. Hu Y. Shi C. Alman B. A. Zhang C. (2022). The role of TMIGD1 as a tumor suppressor in colorectal cancer. Genet. Test. Mol. Biomarkers 26, 174–183. 10.1089/gtmb.2021.0169 35481970

[B37] Qi L. Ding Y. (2017). Screening of tumor suppressor genes in metastatic colorectal cancer. Biomed. Res. Int. 2017, 2769140. 10.1155/2017/2769140 28473981 PMC5394352

[B38] Salmena L. Poliseno L. Tay Y. Kats L. Pandolfi P. P. (2011). A ceRNA hypothesis: The rosetta stone of a hidden RNA language? Cell 146, 353–358. 10.1016/j.cell.2011.07.014 21802130 PMC3235919

[B39] Shen S. Park J. W. Lu Z. x. Lin L. Henry M. D. Wu Y. N. (2014). rMATS: robust and flexible detection of differential alternative splicing from replicate RNA-Seq data. Proc. Natl. Acad. Sci. U. S. A. 111, E5593–E5601. 10.1073/pnas.1419161111 25480548 PMC4280593

[B40] Stamato M. A. Juli G. Romeo E. Ronchetti D. Arbitrio M. Caracciolo D. (2017). Inhibition of EZH2 triggers the tumor suppressive miR-29b network in multiple myeloma. Oncotarget 8, 106527–106537. 10.18632/oncotarget.22507 29290968 PMC5739753

[B41] Sun Z. Ou C. Liu J. Chen C. Zhou Q. Yang S. (2019). YAP1-induced MALAT1 promotes epithelial–mesenchymal transition and angiogenesis by sponging miR-126-5p in colorectal cancer. Oncogene 38, 2627–2644. 10.1038/s41388-018-0628-y 30531836 PMC6484768

[B42] Tang Z. Li C. Kang B. Gao G. Li C. Zhang Z. (2017). Gepia: A web server for cancer and normal gene expression profiling and interactive analyses. Nucleic Acids Res. 45, W98–W102. 10.1093/nar/gkx247 28407145 PMC5570223

[B43] Thorvaldsdottir H. Robinson J. T. Mesirov J. P. (2013). Integrative genomics viewer (IGV): High-performance genomics data visualization and exploration. Brief. Bioinform 14, 178–192. 10.1093/bib/bbs017 22517427 PMC3603213

[B44] Tripathi V. Ellis J. D. Shen Z. Song D. Y. Pan Q. Watt A. T. (2010). The nuclear-retained noncoding RNA MALAT1 regulates alternative splicing by modulating SR splicing factor phosphorylation. Mol. Cell 39, 925–938. 10.1016/j.molcel.2010.08.011 20797886 PMC4158944

[B45] Wang H. Wang L. Zhang G. Lu C. Chu H. Yang R. (2018). MALAT1/miR-101-3p/MCL1 axis mediates cisplatin resistance in lung cancer. Oncotarget 9, 7501–7512. 10.18632/oncotarget.23483 29484127 PMC5800919

[B46] Wang K. Kim M. K. Di Caro G. Wong J. Shalapour S. Wan J. (2014). Interleukin-17 receptor a signaling in transformed enterocytes promotes early colorectal tumorigenesis. Immunity 41, 1052–1063. 10.1016/j.immuni.2014.11.009 25526314 PMC4272447

[B47] Wang W. Zhu Y. Chen X. Jiang G. Shen Z. Qiao Y. (2016). Long noncoding RNA MALAT1 promotes malignant development of esophageal squamous cell carcinoma by targeting β-catenin via Ezh2. Oncotarget 7, 25668–25682. 10.18632/oncotarget.8257 27015363 PMC5041935

[B48] Wang X. Li M. Wang Z. Han S. Tang X. Ge Y. (2015). Silencing of long noncoding RNA MALAT1 by miR-101 and miR-217 inhibits proliferation, migration, and invasion of esophageal squamous cell carcinoma cells. J. Biol. Chem. 290, 3925–3935. 10.1074/jbc.M114.596866 25538231 PMC4326802

[B49] Wei Y. Niu B. (2015). Role of MALAT1 as a prognostic factor for survival in various cancers: A systematic review of the literature with meta-analysis. Dis. markers 2015, 164635. 10.1155/2015/164635 26420912 PMC4572489

[B50] Westphalen C. B. Quante M. Wang T. C. (2017). Functional implication of Dclk1 and Dclk1-expressing cells in cancer. Small GTPases 8, 164–171. 10.1080/21541248.2016.1208792 27458755 PMC5584739

[B51] Wood D. E. Salzberg S. L. (2014). Kraken: Ultrafast metagenomic sequence classification using exact alignments. Genome Biol. 15, R46. 10.1186/gb-2014-15-3-r46 24580807 PMC4053813

[B52] Wu L. Wang X. Guo Y. (2017). Long non-coding RNA MALAT1 is upregulated and involved in cell proliferation, migration and apoptosis in ovarian cancer. Exp. Ther. Med. 13, 3055–3060. 10.3892/etm.2017.4304 28587379 PMC5450566

[B53] Xia H. Chen Q. Chen Y. Ge X. Leng W. Tang Q. (2016). The lncRNA MALAT1 is a novel biomarker for gastric cancer metastasis. Oncotarget 7, 56209–56218. 10.18632/oncotarget.10941 27486823 PMC5302908

[B54] Yang L. Bai H. S. Deng Y. Fan L. (2015). High MALAT1 expression predicts a poor prognosis of cervical cancer and promotes cancer cell growth and invasion. Eur. Rev. Med. Pharmacol. Sci. 19, 3187–3193. 26400521

[B55] Yang L. Lin C. Liu W. Zhang J. Ohgi K. A. Grinstein J. D. (2011). ncRNA- and Pc2 methylation-dependent gene relocation between nuclear structures mediates gene activation programs. Cell 147, 773–788. 10.1016/j.cell.2011.08.054 22078878 PMC3297197

[B56] Zhang B. Arun G. Mao Y. S. Lazar Z. Hung G. Bhattacharjee G. (2012). The lncRNA Malat1 is dispensable for mouse development but its transcription plays a cis-regulatory role in the adult. Cell Rep. 2, 111–123. 10.1016/j.celrep.2012.06.003 22840402 PMC3408587

[B57] Zhang C. Yao K. Zhang J. Wang C. Wang C. Qin C. (2020). Long noncoding RNA MALAT1 promotes colorectal cancer progression by acting as a ceRNA of miR-508-5p to regulate RAB14 expression. Biomed. Res. Int. 2020, 4157606. 10.1155/2020/4157606 33344634 PMC7732393

[B58] Zhang Y. Liu T. Meyer C. A. Eeckhoute J. Johnson D. S. Bernstein B. E. (2008). Model-based analysis of ChIP-seq (MACS). Genome Biol. 9, R137. 10.1186/gb-2008-9-9-r137 18798982 PMC2592715

[B59] Zheng H. T. Shi D. B. Wang Y. W. Li X. X. Xu Y. Tripathi P. (2014). High expression of lncRNA MALAT1 suggests a biomarker of poor prognosis in colorectal cancer. Int. J. Clin. Exp. pathology 7, 3174–3181. PMC4097248 25031737

[B60] Zhou B. Li X. Luo D. Lim D. H. Zhou Y. Fu X. D. (2019). GRID-seq for comprehensive analysis of global RNA-chromatin interactions. Nat. Protoc. 14, 2036–2068. 10.1038/s41596-019-0172-4 31175345 PMC7721247

[B61] Zhu M. Xie J. (2020). LncRNA MALAT1 promotes ulcerative colitis by upregulating lncRNA ANRIL. Dig. Dis. Sci. 65, 3191–3196. 10.1007/s10620-020-06093-w 32026279

