# Supplementary material for: The long non-coding RNA MALAT1 regulates intestine host-microbe interactions and polyposis
Source: Front Cell Dev Biol. 2023 May 30;11:1168693. doi: 10.3389/fcell.2023.1168693 (PMC10265687; doi:10.3389/fcell.2023.1168693)

Supplementary Fig S1. **MALAT1-dependent target validation.**

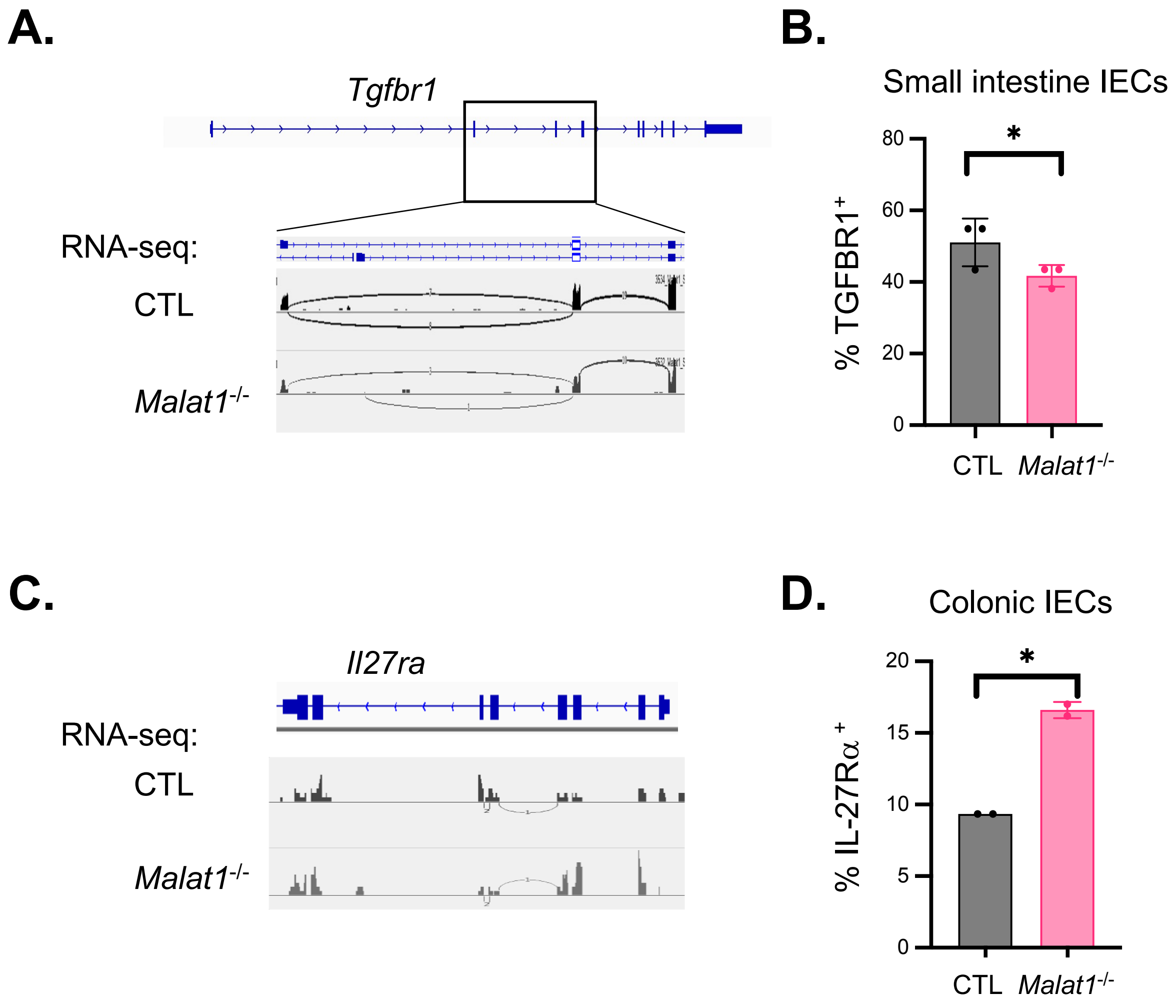

Supplementary Fig S2. **MALAT1-dependent genes involved in response to bacteria.**

A

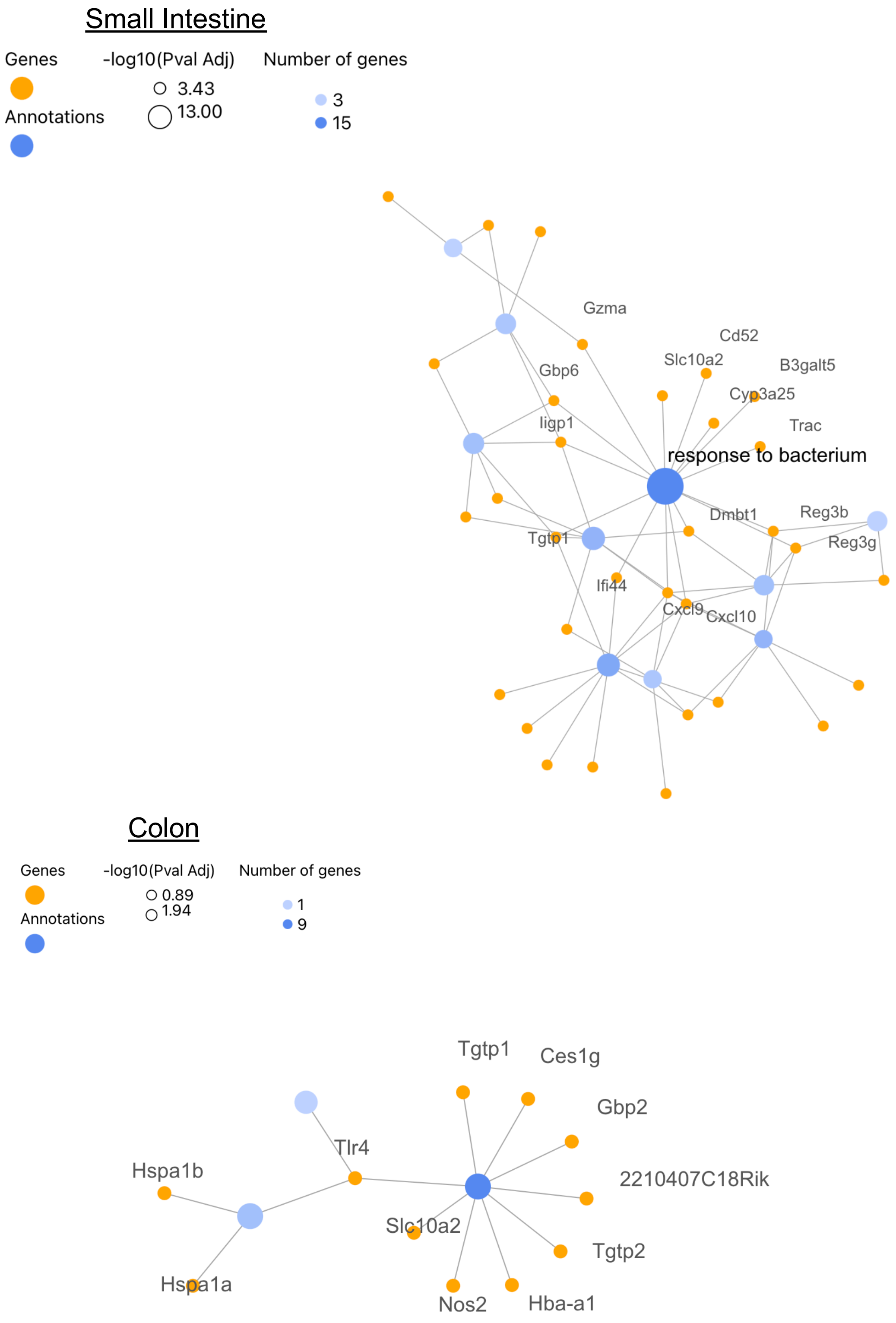

Supplementary Fig S3. **Select small intestine gene expression in the polyposis model.**

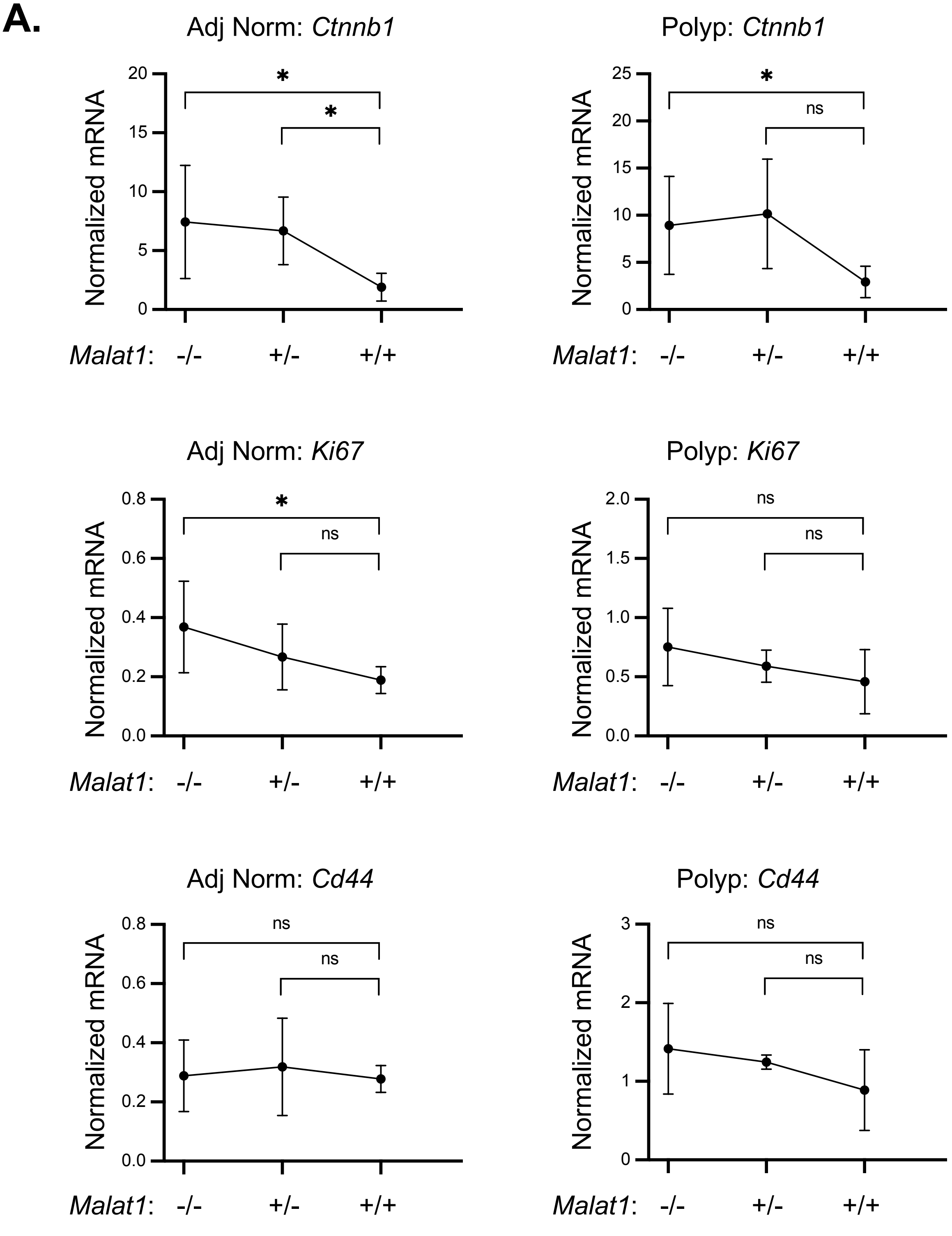

Supplementary Fig S4. **Select colonic gene expression in the polyposis model.**

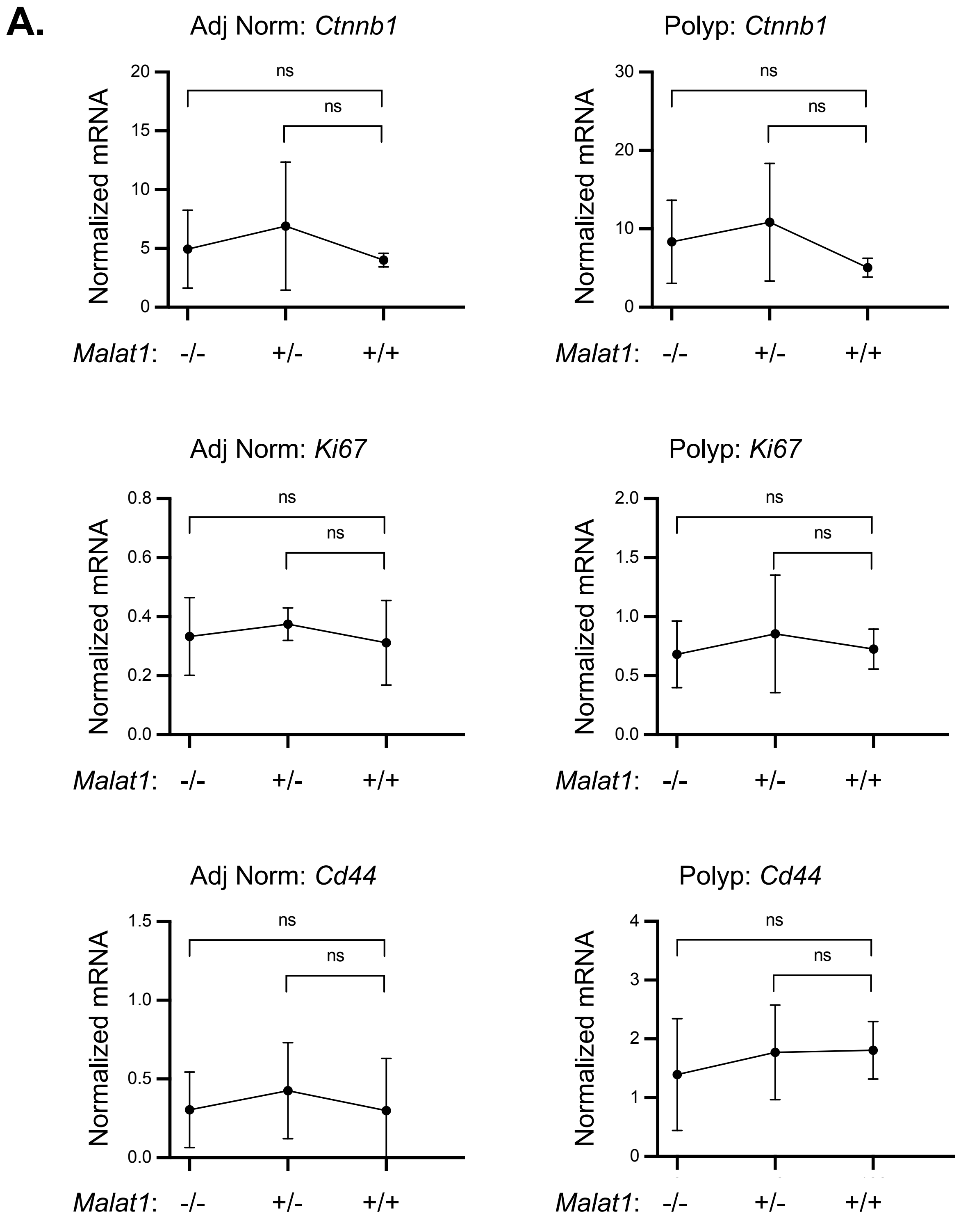

Supplement: Supplementary file 5 [file Image1.pdf]
